# Hemodynamic consequences of pressure-flow curve gradient variations in continuous-flow ventricular assist devices

**DOI:** 10.3389/fphys.2025.1730883

**Published:** 2026-01-12

**Authors:** Yuzhuo Yang, Zhenyu Wang, Zipeng Xie, Shunzhou Yu, Liang Zou

**Affiliations:** 1 ShenZhen Core Medical Technology CO, LTD, Shenzhen, China; 2 Harbin Institute of Technology, Harbin, China; 3 Chinese Academy of Medical Sciences and Peking Union Medical College Fuwai Hospital, Beijing, China

**Keywords:** heart FaiIure, hemodynamic, H-Q curve, lumped parameter model, ventricle assist device

## Abstract

**Background:**

Continuous-flow ventricular assist devices (VADs) have been widely adopted in clinical practice for the treatment of heart failure, but the effect of their non-pulsatile blood flow on microvascular circulation is still debated. Although VADs with a flatter H-Q curve are known to produce greater pulse pressure (PP), other hemodynamic performances have not been systematically compared and analyzed.

**Methods:**

This study employed a lumped-parameter cardiopulmonary circulation numerical model to compare the hemodynamic responses of two continuous-flow centrifugal pumps: the Corheart 6 (flatter H-Q curve) and the HeartMate 3 (steeper H-Q curve). Comparisons were conducted across four distinct clinical scenarios: left heart failure, right heart failure, myocardial recovery and acute preload shifts. A quantitative assessment focused on arterial PP, peripheral organ perfusion, ventricular unloading, pump suction risk, and pump thrombosis risk.

**Results:**

At the same average pump flow, pumps with a flatter H-Q curve, because of their higher sensitivity to preload, generated higher pump flow pulsatility and greater arterial PP, thereby creating hemodynamic conditions that may theoretically reduce risks associated with flow stasis. However, their ventricular unloading and peripheral organ perfusion were slightly inferior. When pump speed was increased, these pumps achieved ventricular unloading and peripheral organ perfusion comparable to those with steeper H-Q curves while simultaneously yielding even higher arterial PP. In contrast to the static condition, during dynamic events such as acute preload reduction caused by postural changes, VADs with a flatter H-Q curve are better able to maintain systemic perfusion pressure. When applied in right heart failure, right atrium implantation yields superior right ventricular unloading but lower pump flow pulsatility of both pumps.

**Conclusion:**

The findings provide references for VAD developers and clinicians for the optimal design and utilization of blood pumps with different H-Q characteristics.

## Introduction

The earliest ventricular assist devices (VADs) employed a pulsatile flow mechanism, mimicking physiological cardiac contraction and relaxation by synchronizing periodic volume changes with heartbeats. However, their substantial size and low reliability limited widespread clinical adoption (Feller et al., 2007; Garatti et al., 2008; Kamdar et al., 2009). In contrast, continuous-flow VADs have become the primary choice for long-term circulatory support due to their smaller size, higher reliability, and fewer adverse events (Caccamo et al., 2011; Teutebe et al., 2020; Cusimano et al., 2021). Nevertheless, some studies have pointed out that their non-pulsatile flow can have detrimental effects on systemic and microvascular circulation, including inhibition of endothelial nitric oxide synthase, which can lead to vascular endothelial dysfunction and increased arterial stiffness (Bartoli et al., 2010; Khambadkone et al., 2003). Under prolonged continuous-flow support, this could elevate the risk of complications such as arteriovenous malformations and gastrointestinal bleeding.

To better approximate normal physiological characteristics, certain continuous-flow VADs have been designed with flatter H-Q curves (Fang P et al., 2023; Motomura et al., 2020), enabling them to generate greater pump flow pulsatility and pulse pressure (PP). Bartoil et al. investigated the hemodynamic response to continuous-flow (CF) and pulsatile-flow (PF) pumps in animal models and demonstrated that CF pumps impair the physiological pulsatility of hemodynamics during ventricular unloading, whereas PF pumps maintain more normative physiological values (Bartoli et al., 2010). The literature has established that reduced physiological pulsatility adversely affects both the macro- and micro-circulation, potentially causing vascular stiffening due to the suppression of endothelial nitric oxide synthase activity (Khambadkone et al., 2003). Several studies have investigated the impact of H-Q curve slope in continuous-flow VADs. Sénage et al. (2014) used an experimental model to simulate and compare the hemodynamic characteristics of an axial-flow pump (HeartMate II, HM II) and a centrifugal pump (VentrAssist, VTA). Their findings showed that, under the same total cardiac output (CO), support with HM II resulted in higher mean arterial pressure, lower left atrial pressure, and higher right atrial pressure, but also carried a higher risk of ventricular suction, which is consistent with the conclusion of study (Giridharan et al., 2015). Eleuteri et al. (2012) investigated differences in myocardial reverse remodeling between axial-flow and centrifugal pumps by measuring changes in left ventricular end-diastolic diameter (LVEDD) before and after implantation. The results indicated that patients supported by axial-flow pumps had smaller LVEDD and greater reductions in the biomarker associated with reverse remodeling. Graefe et al. (2019) found that pumps with flatter H-Q curves achieved higher peak flow rates and thus providing better ventricular unloading during exercise. Telyshev et al. (2019) used a lumped parameter model to compare the hemodynamic effects between VADs. At the same average pump flow, HeartWare demonstrated superior ventricular unloading, evidenced by a smaller ventricular stroke volume. This result differed from Sénage’s findings because HeartWare exhibited a flatter H-Q curve in low-flow regions and a steeper curve in high-flow regions compared to HM II.

These studies primarily focused on comparing the impacts of different H-Q curves between axial and centrifugal pumps on key hemodynamic parameters in patients with LHF. However, they did not extensively explore or quantitatively discuss the impact of parameters such as ventricular volume, ventricular pressure, arterial pressure, and pump flow on clinical safety (e.g., suction/thrombosis risk) and effectiveness (e.g., organ perfusion). In addition, VADs are also clinically used for treating right heart failure (RHF). Clinicians sometimes prefer right atrium (RA) implantation over right ventricle (RV) implantation, but clinical outcomes showed no significant differences in pump thrombosis rates or gastrointestinal bleeding (Maynes et al., 2020). However, a comparative analysis of the hemodynamics of these two implantation methods is lacking. Furthermore, the hemodynamic consequences of myocardial recovery and dynamic condition have not been systematically analyzed across different H-Q curve characteristics.

This paper aims to systematically compare and quantitatively evaluate the hemodynamic consequences of two representative, mainstream third-generation magnetic levitation centrifugal VADs—the Corheart 6 (flatter H-Q curve) and the HeartMate 3 (steeper H-Q curve)—across four key clinical application scenarios to inform optimal VAD design and utilization:

1. LHF with left ventricle (LV) implantation.
2. RHF with RA or RV implantation.
3. Myocardial recovery in both LHF and RHF scenarios.
4. Acute preload shifts.

Through a lumped parameter numerical hemodynamic model, this study will quantify the impact of different H-Q slopes on key clinical safety and efficacy indicators, including arterial pulse pressure (PP_ao_), peripheral organ perfusion (MAP), ventricular unloading (ESP, EDV), pump suction risk (EDV, PI_VAD_), and pump thrombosis risk (PI_VAD_). This systematic and quantitative comparison is intended to fill the gaps in the existing literature and provide direct reference for current clinical practice.

## Methods

### Mathematical model of cardiovascular system

**FIGURE 1:**
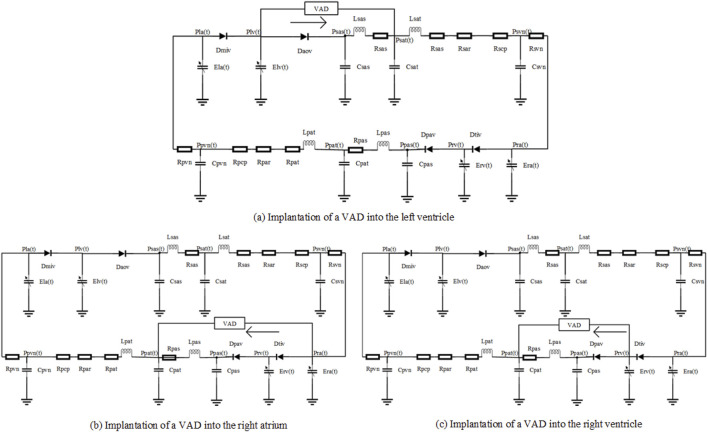
Lumped parameter models of circulatory system with VAD implantation in different heart chambers. In the numerical model, D_miv_, D_aov_, D_tiv_ and D_pav_ represent mitral, aortic, tricuspid, and pulmonary valves, respectively; R, L and C denote resistance, inertance and compliance, P and Q denote pressure and flow rate, lv, la, rv, and ra denote left ventricle, left atrium, right ventricle, and right atrium; sas, sat, pas, and pat represent the aortic sinus, aorta, pulmonary sinus, and pulmonary artery, respectively; sar, scp, par, and pcp represent systemic arteries, systemic capillaries, pulmonary arterioles, and pulmonary capillaries; and svn and pvn denote systemic veins and pulmonary veins.

A numerical model of the cardiovascular circulation system was employed for simulation experiments, with its construction referencing existing literature (Liu et al., 2020; Korakianitis and Shi, 2006; Hall and Hall, 2011; Westerhof et al., 2009; Pant et al., 2018). These data and waveforms in our article are all consistent with hemodynamic information in the Textbook of Medical Physiology and Guyton and Hall Textbook of Medical Physiology (Liu et al., 2020). As shown in Figure 1, the model consists of the left heart, right heart, systemic circulation, and pulmonary circulation. The arterial system is modeled using the Windkessel model. In this model, all pressure variables are defined as transmural pressures (the difference between internal and external chamber pressures), and the potential physiological effects of pericardial constraint on the heart chambers are not considered.

The heart is equivalently represented as a chamber with time-varying elastance during the cardiac cycle (Suga and Sagawa, 1974; Karimov et al., 2020; Simaan et al., 2009; Stergiopulos et al., 1996). Ventricular elastance is defined as the ratio of ventricular pressure to volume, which is the reciprocal of capacitance:

$$E_{lv}\left(t\right)=\frac{1}{C_{lv}\left(t\right)}=\frac{P_{lv}\left(t\right)}{V_{lv}\left(t\right)-V_{lv,0}}$$ (1)

$$E_{rv}\left(t\right)=\frac{1}{C_{rv}\left(t\right)}=\frac{P_{rv}\left(t\right)}{V_{rv}\left(t\right)-V_{rv,0}}$$ (2)

In Equations 1, 2, *E*_*lv*_(*t*) and *E*_*rv*_(*t*) are the time-varying elastances of the LV and RV, and *C*_*lv*_(*t*) and *C*_*rv*_(*t*) are the time-varying compliances of the LV and RV. *P*_*lv*_(*t*) and *P*_*rv*_(*t*) and *V*_*lv*_(*t*) and *V*_*rv*_(*t*) are the pressures and volumes of the LV and RV, respectively. *V*_*lv,0*_(*t*) and *V*_*rv,0*_(*t*) are theoretical volumes of LV and RV at zero pressure, respectively.

$$E_{lv}\left(t\right)=\left(E_{\max,lv}-E_{\min,lv}\right)E_{n,lv}\left(t_{n}\right)+E_{\min,lv}$$ (3)

$$E_{rv}\left(t\right)=\left(E_{\max,rv}-E_{\min,rv}\right)E_{n,rv}\left(t_{n}\right)+E_{\min,rv}$$ (4)

In Equations 3, 4, *E*_*max* *,lv*_ and *E*_*min* *,lv*_ are left ventricular end-systolic elastance and left ventricular end-diastolic elastance, respectively, and *E*_*max* *,rv*_ and *E*_*min* *,rv*_ are right ventricular end-systolic elastance and right ventricular end-diastolic elastance, respectively. *E*_*n,lv*_ *(t*_*n*_ *)*, *E*_*n,rv*_ *(t*_*n*_ *)* are normalized ‘double-Hill’ function expressions, which define the steepness and shape of the curve. The same normalized elastance formula is used for both ventricles since the activities of the LV and RV are synchronized.

## Valve

The heart valves were simplified as ideal diodes in the model to ensure unidirectional blood flow (Korakianitis and Shi, 2006). While this approach is standard practice in lumped-parameter models, its primary limitation is the inability to simulate valve regurgitation. Nevertheless, this simplification remains effective for assessing the relative hemodynamic impact of VAD support on ventricular unloading. The opening and closing of the aortic valve is controlled by the pressure difference between the LV and the aorta:

$$Q_{aov}=\left\{\begin{array}{c} 350\cdot {AR}_{aov}\cdot \sqrt{P_{lv}-P_{sas}},\;P_{lv}\geq P_{sas}\\ 350\cdot {AR}_{aov}\cdot \sqrt{P_{sas}-P_{lv}},\;P_{lv}<P_{sas} \end{array}\right.$$ (5)

$$Q_{miv}=\left\{\begin{array}{c} 400\cdot {AR}_{miv}\cdot \sqrt{P_{la}-P_{lv}},\;P_{la}\geq P_{lv}\\ 400\cdot {AR}_{miv}\cdot \sqrt{P_{lv}-P_{la}},\;P_{la}<P_{lv} \end{array}\right.$$ (6)

where *Q*_*aov*_ and *Q*_*miv*_ are the flow rates through the aortic and mitral valves, *P*_*lv*_, *P*_*sas*_, and *P*_*la*_ are the pressures of the LV, aortic sinus, and left atrium, respectively, and the valve opening parameters *AR*_*aov*_ and *AR*_*miv*_ switch between 0 and 1:

$${AR}_{aov}=\left\{\begin{array}{c} 1,\;P_{lv}\geq P_{sas}\\ 0,\;P_{lv}<P_{sas} \end{array}\right.$$ (7)

$${AR}_{miv}=\left\{\begin{array}{c} 1,\;P_{la}\geq P_{lv}\\ 0,\;P_{la}<P_{lv} \end{array}\right.$$ (8)

### Ventricular assist device

**FIGURE 2:**
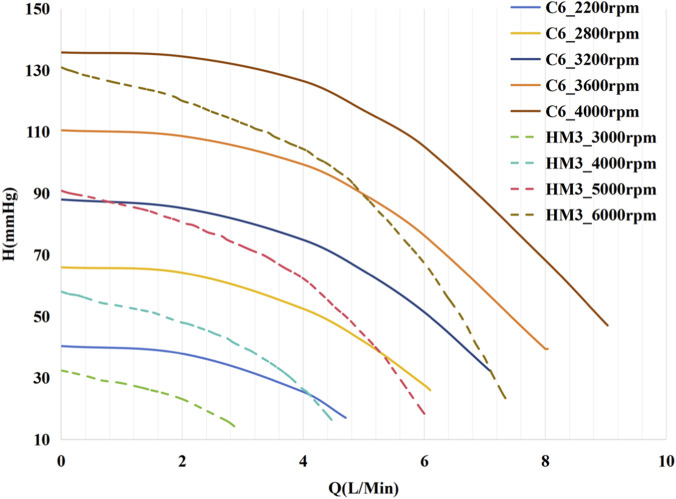
H–Q curves of C6 and HM3 continuous-flow centrifugal pumps at different rotational speeds (operating speed ranges: 2200–4300 rpm for C6 (Fang P et al., 2023), 3000–9000 rpm for HM3 (Zayat et al., 2019)).

This study compared the hemodynamic effects of C6^9^ and HM3 (Meissner et al., 2023; Abbott, 2025). As depicted in Figure 2, the C6’s H-Q curve consistently remains flatter than that of the HM3 across the entire operational flow range. The H-Q characteristic curve of the pump is expressed using the following equation. The fitting process was performed using the least squares method, and the coefficient of determination *R* ^2^ was used to evaluate the goodness of fit to the original data:

$$H=a*Q^{2}+b*Q*w+c*w^{2}$$ (9)

Where *H* is the pump head (mmHg), *Q* is the pump flow rate (L/min), *w* is the pump rotational speed (rpm), and *a*, *b*, and *c* are coefficients related to the rotary pump and obtained through least-squares fitting. Table 1 presents the fitting coefficients for the characteristic curves of the two pumps.

**TABLE 1:** Pump H-Q characteristic curve fitting parameters.

Pump	*a*, mmHg min/L	*b*, mmHg min^2^/L^2^	*c*, mmHg min^2^/L^2^	*R* ^2^
C6	−1.552	1.125e-03	8.319e-06	0.9988
HM3	−2.431	9.467e-04	3.399e-06	0.9954

### Simulation cases

The different simulation experiments were conducted in MATLAB Simulink. The ode45 solver was employed for solving the system of equations. The time step was set to 0.001 s. All simulation durations were set to 60 s with the system achieving a stable periodic solution after approximately 10 s. The stable results from the 58 s to the 60 s were selected for analysis across all simulated conditions.

Table 2 lists the key parameters used in the numerical model, all parameters of the hemodynamic model were set according to published literature (Liu et al., 2020; Korakianitis and Shi, 2006; Hall and Hall, 2011; Westerhof et al., 2009; Pant et al., 2018), and identifiability and robustness of the model has been examined, ensuring that the simulated hemodynamic results aligned with physiological characteristics. The R elements represent the microvascular resistance of the vascular system, and the C elements represent the elasticity or volume buffering capacity of the vascular walls. The combined action of the R and C elements constitutes the Windkessel effect, which determines the pulsatility of the arterial pressure waveform. The severity of heart failure (HF) was then modulated by adjusting the maximum ventricular elastance (*E*_max_), with a lower elasticity indicating more severe HF. To isolate the hemodynamic effects of the different H-Q curve characteristics and avoid different volumetric support as a confounding factor, the RPM of both pumps were adjusted to achieve an identical mean pump flow.

**TABLE 2:** Key model parameters inital value.

Parameter	Value	Unit
Rsat	0.0398	mmHg·s/mL
Lsat	0.0005	mmHg·s^2^/ml
Csat	0.08	mL/mmHg
Elv,max	2.19	mmHg/mL
Elv,min	0.04	mmHg/mL
Ela,max	0.25	mmHg/mL
Ela,min	0.15	mmHg/mL
Erv,max	0.84	mmHg/mL
Erv,min	0.04	mmHg/mL
Era,max	0.25	mmHg/mL
Era,min	0.15	mmHg/mL
Vlv,0	450	ml
Vrv,0	400	ml
Rpat	0.005	mmHg·s/mL
Lpat	0.0005	mmHg·s^2^/ml
Cpat	0.6	mL/mmHg

R, L and C denote resistance, inertance and compliance, E denote elasticity, lv, la, rv, and ra denote left ventricle, left atrium, right ventricle, and right atrium; sat and pat represent the aorta and pulmonary artery, respectively.

Case 1: Left heart failure, VAD inflow cannula implanted in the left ventricle.

The numerical model is established by adjusting *E*_*max,lv*_ from a healthy value of 2.19 mmHg/mL to 1.25 mmHg/mL, with *E*_*max,rv*_ from a healthy value of 0.84 mmHg/mL to 0.55 mmHg/mL. According to the literature (Desai et al., 2011), mild RHF is a common complication of LHF.

Case 2: Right heart failure with healthy left heart, VAD inflow cannula individually implanted in the right ventricle and right atrium.

The numerical model adjusts Emax,rv from a healthy value of 0.84 mmHg/mL to 0.3 mmHg/mL.

Case 3: Myocardial recovery.

The elasticity of ventricular was increased in both Case 1 and Case 2. Specifically, *E_max,lv_* was increased from 1.25 mmHg/mL to 2.0 mmHg/mL with the *E_max,rv_* from 0.55 mmHg/mL to 0.84 mmHg/mL in Case 1 and *E_max,rv_* was increased from 0.3 mmHg/mL to 0.6 mmHg/mL in Case 2, indicating a partial recovery of cardiac function.

Case 4: Acute Preload Change Simulating Postural Hypotension.

The systemic venous compliance Csvn was acutely increased from 20.5 mmHg·s/mL to 30 mmHg·s/mL. Based on previously published literature (Tyberg and Douglas, 1996), this systemic adjustment of the Csvn parameter serves to simulate the redistribution of blood volume between the central and peripheral circulations caused by postural changes (e.g., transition from supine to upright position), thereby inducing an acute change in cardiac preload.

The following variables were evaluated in these test cases:

*LVESP, RVESP*: Left, right ventricular end-systolic pressure, mmHg.

*LVEDP, RVEDP*: Left, right ventricular end-diastolic pressure, mmHg.

*LVESV, RVESV*: Left, right ventricular end-systolic volume, mL.

*LVEDV, RVEDV*: Left, right ventricular end-diastolic volume, mL.

P-V loop: Ventricular pressure-volume loop.

*SPAP*: Systolic pulmonary artery pressure, mmHg.

*DPAP*: Diastolic pulmonary artery pressure, mmHg.

*SAP*: Systolic aortic pressure, mmHg.

*DAP*: Diastolic aortic pressure, mmHg.

*PP_ao_*: Aortic pulse pressure, mmHg, calculated as follows:

$${PP}_{ao}=SAP-DAP$$ (10)

*PP_pa_*: Pulmonary aortic pulse pressure, mmHg, calculated as follows:

$${PP}_{pa}=SPAP-DPAP$$ (11)

*MAP*: Mean aortic pressure, mmHg, calculated as follows:

$$MAP=\frac{2\cdot DAP+SAP}{3}$$ (12)

*MPAP*: Mean pulmonary artery pressure, mmHg, calculated as follows:

$$MPAP=\frac{2\cdot DPAP+SPAP}{3}$$ (13)

CCP: Coronary perfusion pressure, mmHg, using pressure gradient to assess (Nguyen et al., 2018).

CCP=DAP−LVEDP (14)

*Q_AV_*: Aortic valve real-time flow, L/min.

*Q_PV_*: Pulmonary valve real-time flow, L/min.

*Q_AV,AVG_*: Mean aortic valve flow over one cardiac cycle, L/min, calculated as follows:

$$Q_{AV,AVG}=\int_{0}^{T}Q_{AV}\;dt$$ (15)

where *T* is the duration of cardiac cycle, s, *Q_VAD_* is VAD real-time flow, L/min, and *Q_VAD,AVG_* is mean pump flow over one cardiac cycle, L/min, calculated as follows:

$$Q_{VAD,AVG}=\int_{0}^{T}Q_{VAD}\;dt$$ (16)

*PI_VAD_* is pump flow pulsatility index, physiologically reflects the pulsatility of VAD blood flow and serve as a surrogate marker for native heart contribution and systemic microcirculatory health, calculated as follows:

$${PI}_{VAD}=\frac{Q_{VAD,\mathrm{MAX}}-Q_{VAD,\mathrm{MIN}}}{Q_{VAD,AVG}}$$ (17)

*CO* is total cardiac output, L/min, calculated as follows:

$$CO=Q_{AV,AVG}+Q_{VAD,AVG}$$ (18)

## Results

Case 1 Left heart failure, VADs implanted in the left ventricle.

**FIGURE 3:**
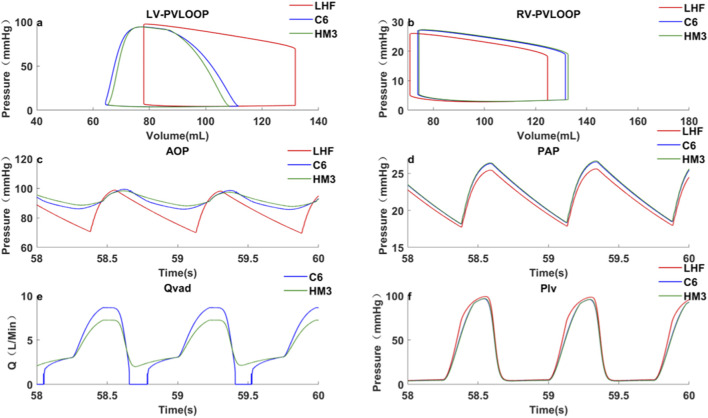
LHF, VAD implantation in the ventricle, hemodynamic characteristics under pump support and baseline conditions: **(a)** Left ventricular pressure-volume loop; **(b)** Right ventricular pressure-volume loop; **(c)** Aortic pressure curve; **(d)** Pulmonary artery pressure curve; **(e)** Pump flow curve; **(f)** Left ventricular pressure curve; LHF represents left heart failure baseline condition without VAD support; C6 represents the condition under Corheart 6 support; HM3 represents the condition under HeartMate 3 support.

The speed of the pumps was adjusted to achieve the same *Q_VAD,AVG_* of 4.3 L/min, approximating full support flow. This corresponded to a C6 speed of 3200 rpm and an HM3 speed of 5200 rpm. Results were shown in Table 3 and Figure 3. Notably, the simulated hemodynamics of the HeartMate 3 in LHF scenario (5200 rpm, MAP 89.7 mmHg, pump flow 4.3 L/min) demonstrated close agreement with reported clinical measurements (Uriel et al., 2017; Montalto et al., 2019), confirming the model’s physiological fidelity.

In terms of perfusion, at the same average pump flow rate (4.3 L/min), the HM3, which has a steeper H-Q curve, achieved an *MAP* of 89.7 ± 0.3 mmHg and a CCP of 83.3 ± 0.5 mmHg. These values were 0.7 mmHg (0.8%) and 1.8 mmHg (2.2%) higher than those achieved by the C6 (*MAP*: 89.0 ± 0.2 mmHg; CCP:81.5 ± 0.4 mmHg), respectively, indicating superior organ perfusion and coronary perfusion.

Regarding ventricular unloading, the PV loop (Figure 3a) demonstrates comparable LVESP (C6: 93.8 mmHg; HM3: 93.9 mmHg), yet the HM3 exhibits a slightly lower LVEDV (108.1 mL versus 110.0 mL). Mechanistically, this difference is attributed to the HM3’s steeper H-Q curve (with a higher *dH/dQ* ratio), which resulted in a smaller variation in pump flow from systole to diastole. While C6 generated a higher peak systolic flow (8.7 L/min compared to 7.2 L/min for HM3), its diastolic flow was nearly zero. In contrast, HM3 sustained a diastolic flow of 2.4 L/min, which contributed to the low LVEDV and more effective ventricular unloading. Conversely, regarding arterial PP, the C6 (with a flatter H-Q curve) was 11.6 ± 0.4 mmHg, which was significantly higher than that of the HM3 (8.6 ± 0.3 mmHg).

In terms of other hemodynamic-related complications, the C6 maintained a higher LVEDV and exhibited greater *PI_VAD_*, making it less prone to ventricular suction during diastole and a lower risk of pump thrombosis (Fang P et al., 2022). Although third-generation magnetic levitation pumps rarely experience intrinsic pump thrombosis (Nascimbene et al., 2024), there remains a possibility of externally introduced thrombi or tissue causing pump thrombosis during support. Greater pump flow pulsatility can more effectively flush near-wall flow passages and low-velocity regions within the pump (Fang P et al., 2022), thereby reducing the risk of spontaneous intrapump thrombosis or lodging and subsequent thrombosis of exogenous material within the pump.

**TABLE 3:** Hemodynamic and pump parameters: Baseline and post-LVAD implantation in LHF.

Case	*RPM* */rpm*	*ΔCO* */L/min*	*MAP* */mmHg*	*PPao* */mmHg*	*CCP* */mmHg*	*LVESP* */mmHg*	*LVEDV* */mL*	*Q*_*av,AVG*_ */L/min*	*Q*_*VAD,AVG*_ */L/min*	*Q*_*VAD*_ *(PI*_*VAD*_ *)* */L/min*	*MPAP* */mmHg*
Basic	0	0	78	28.2	64.6	97.3	131.7	3.5	0	0	20.7
C6	3200	0.9	89	11.6	81.5	93.8	110	0.1	4.3	0–8.7 (2.0)	21.6
HM3	5200	1.0	89.7	8.6	83.3	93.9	108.1	0.2	4.3	2.4–7.2 (1.1)	21.7
C6-recovery	3200	1.4	97.7	23.1	86.8	110.9	110.9	1.6	3.3	0–8.7 (2.6)	24.0
HM3-recovery	5200	1.5	98.9	19.9	88.9	111.3	107.9	1.7	3.3	0–7.3 (2.2)	24.1

C6: Corheart 6; HM3: HeartMate 3; C6-recovery: Corheart 6 support after myocardial recovery; HM3-recovery: HeartMate 3 support after myocardial recovery; *ΔCO*, denotes the increment of cardiac output compared with the baseline (L/min); *MAP*: mean aortic pressure (mmHg); *PPao:* aortic pulse pressure (mmHg); *CCP*: coronary perfusion pressure (mmHg); *LVESP*: left ventricular end-systolic pressure (mmHg); *LVEDV*: left ventricular end-diastolic volume (mL); *Q*_*av,AVG*_: mean aortic valve flow over one cardiac cycle (L/min); *Q*_*VAD,AVG*_: mean pump flow over one cardiac cycle (L/min); *Q*_*VAD*_ *(PI*_*VAD*_ *)*: pump real-time flow (pump flow pulsatility index); *MPAP*: mean pulmonary artery pressure (mmHg).

Case 2 Right heart failure with healthy left heart, VADs individually implanted in the right atrium or right ventricle.

**FIGURE 4:**
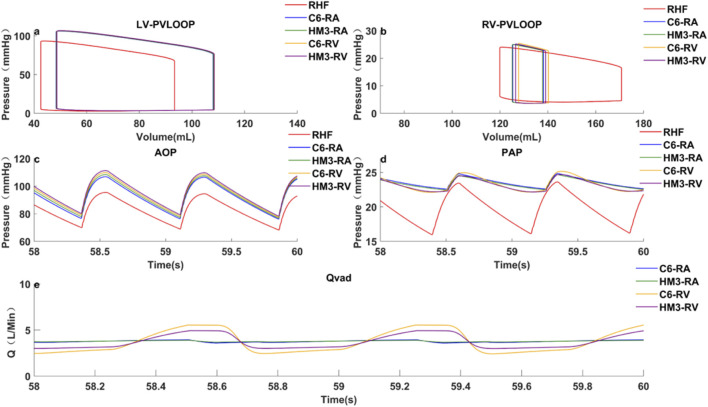
RHF with RA/RV VAD implantation, hemodynamic characteristics under pumps support and baseline conditions. **(a)** left ventricular pressure-volume loop; **(b)** Right ventricular pressure-volume loop; **(c)** Aortic pressure curve; **(d)** Pulmonary artery pressure curve; **(e)** Pump flow curve; RHF represents RHF baseline condition without VAD support; C6-RA/RV represents the RA/RV under Corheart 6 support; HM3-RA/RV represents the RA/RV under HeartMate 3 support.

The VAD inflow cannula was connected to RA, and the pumps were adjusted to the same *Q*_*VAD,AVG*_ of 3.7 L/min. Results were shown in Table 4; Figure 4.

Hemodynamic parameter results revealed the following: *MAP*, *MPAP*, *PP*_*pa*_, *RVESP*, and *RVEDP* were comparable for both pumps, indicating similar organ perfusion, arterial PP and ventricular unloading. Concerning other hemodynamic-related complications, the C6 exhibited greater *PI*_*VAD*_, leading to better pump washout and a lower likelihood of suction events.

Subsequently, the VAD inflow was relocated from RA to RV. The pumps were again adjusted to the same *Q*_*VAD,AVG*_ of 3.7 L/min. The results, presented in Table 4; Figure 4, show that both pumps have comparable organ perfusion, and C6 shows less effective right ventricular unloading and a greater *PP*_*pa*_. Because the periodic variation of right ventricular pressure was greater than right atrial pressure, when implanted in RV, the diastolic pump flow of C6 was lower than that of HM3, thereby reducing the right ventricular unloading. In terms of other hemodynamic-related complications, the C6 demonstrated greater flow pulsatility and was less prone to pump thrombosis and suction events.

**TABLE 4:** Hemodynamic and pump parameters: Baseline and post-RVAD (RA/RV) implantation in RHF with healthy left heart.

Case	Pump	*RPM* */rpm*	*ΔCO* */L/min*	*MAP* */mmHg*	*RVESP* */mmHg*	*RVEDV* */mL*	*RVESV* */mL*	*Qpv,*_*AVG*_ */L/min*	*Q*_*VAD,AVG*_ */L/min*	*Q*_*VAD*_ *(PI*_*VAD*_ *)* */L/min*	*MPAP* */mmHg*	*PP*_*pa*_ */mmHg*
Basic	-	0	0	75.8	24.0	170.9	120	3.60	0	0	18.8	7.5
RA	C6	2,250	1.26	85.6	25.0	138.2	125.4	1.16	3.70	3.56–3.93(0.09)	23.5	2.3
	HM3	3850	1.25	85.6	25.0	138.1	125.3	1.15	3.70	3.64–3.87(0.06)	23.5	2.3
RV	C6	2040	1.22	85.7	25.5	140.7	128.1	1.12	3.70	2.32–5.54(0.87)	23.5	3.1
	HM3	3530	1.22	85.7	25.3	139.6	127.0	1.12	3.70	2.92–4.93(0.54)	23.5	2.7
RA-recover	C6	2,250	1.59	96	29.7	105.3	74.3	2.26	2.93	2.48–3.49(0.34)	26.3	4.9
	HM3	3850	1.71	96.7	29.7	102.5	75.4	2.08	3.23	3.02–3.54(0.16)	26.6	4.5
RV-recover	C6	2040	1.44	96.4	30.9	109.5	77.4	2.29	2.75	0–5.58(2.03)	26.4	6.6
	HM3	3530	1.72	97.5	30.4	102.8	76.1	1.96	3.36	2.00–4.95(0.88)	26.9	5.1

*ΔCO*, denotes the increment of cardiac output compared with the baseline (L/min); *MAP*: mean aortic pressure (mmHg); *RVESP*: right ventricular end-systolic pressure (mmHg); *RVEDV*: right ventricular end-diastolic volume (mL); *RVESV*: right ventricular end-systolic volume (mL); *Q*_*pv,AVG*_: mean pulmonary valve flow over one cardiac cycle (L/min); *Q*_*VAD,AVG*_: mean pump flow over one cardiac cycle (L/min); *Q*_*VAD*_ *(PI*_*VAD*_ *)*: pump real-time flow (pump flow pulsatility index); *MPAP*: mean pulmonary artery pressure (mmHg); *PPpa:* pulmonary pulse pressure (mmHg).

Case 3 Myocardial recovery.

1. Left ventricular function recovery

Hemodynamic and pump flow characteristics are presented in Table 3, which shows increased *CO, MAP* and *PP*_*ao*_ while *Q*_*VAD,AVG*_ decreased. C6’s *LVESV* was slightly lower than HM3’s, whereas its *LVEDV* was slightly higher. This is because C6 has a greater pump flow during systolic phase and a slightly smaller pump flow during diastolic phase.

1. Right ventricular function recovery

The results were presented in Table 4. It was observed that pumps with varying H-Q curves exert distinct hemodynamic impacts based on their placement. For C6, RA implantation resulted in lower *RVEDV* and *RVESV* compared to RV implantation. This occurred because as right heart function recovers, the pressure gradient between DPAP and RVEDP is greater than that between DPAP and right atrial diastolic pressure. This leads to reduced diastolic pump flow during RV implantation. In contrast, for HM3, with its steeper H-Q curve, changes in pressure gradient have a less impact on pump flow.

Case 4 Acute Preload Change Simulating Postural Hypotension.

**FIGURE 5:**
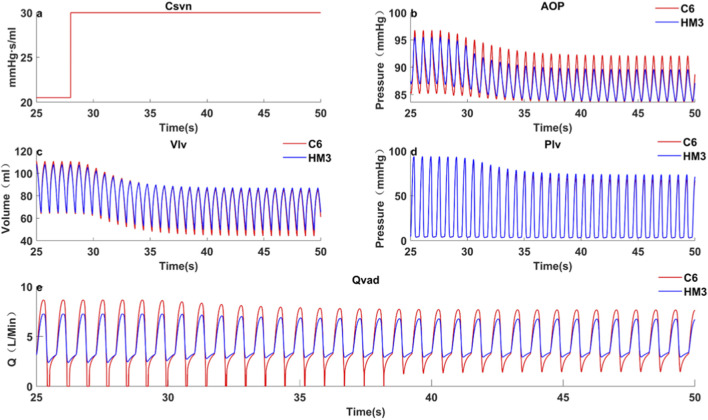
Comparison of hemodynamics between C6 and HM3 under acute preload change due to postural shift.

An abrupt increase in *C*_*svn*_ at 28s (simulating a decrease in preload caused by postural change) resulted in a greater volume of blood being sequestered in the veins, thus reducing venous return. As shown in Figure 5, this reduction in venous return led to a sharp decrease in *LVEDV* for both pumps (C6: Δ22.87 mL; HM3: Δ20.71 mL), suggesting an increased risk of suction for both, with C6 showing a marginally greater *LVEDV* drop. The decrease in preload was followed by a reduction in aortic pressure. As the reduced aortic pressure lowered the hydraulic head across the pump, distinct flow responses were observed between the two devices. Owing to its flatter H-Q characteristic, C6 exhibited an obvious increase in diastolic pump flow. In contrast, HM3 showed a more limited diastolic flow response. Despite the reduction in *LVEDV*, the C6 pump’s flatter H-Q curve resulted in a more pronounced increase in its diastolic flow. This compensatory mechanism enabled C6 to maintain systemic perfusion more effectively during the acute preload change, evidenced by a smaller decrease in *MAP* (C6: 89.1 mmHg–86.5 mmHg, Δ2.6 mmHg; HM3: 90 mmHg–85.7 mmHg, Δ4.3 mmHg).

## Discussion

Under the same average pump flow, C6, with its flatter H-Q curve, demonstrates greater sensitivity to changes in preload, generating stronger pump flow pulsatility and higher arterial PP(Pulse pressure). These hemodynamic characteristics may partially mitigate the adverse stimuli associated with continuous flow, which have been shown in previous studies to contribute to endothelial dysfunction. Moreover, under normal HR conditions (60–100 bpm), diastole is approximately twice as long as systole. Consequently, with the same average pump flow, the C6 directs more of its flow output toward systole compared to the HM3. As a result, the C6 shows slightly inferior performance in terms of peripheral organ perfusion (evaluated by *MAP(Mean aortic pressure)*) and ventricular unloading. However, when HR changes significantly, such as during exercise or arrhythmias, this difference may decrease or even be reversed. This is because the proportion of diastole in the cardiac cycle may drop significantly (potentially below 50%). Thus, the overall hemodynamic response primarily depends on the relative duration and actual pump flow within systole and diastole.

In RHF application scenario, a high pump flow can be generated even at low rotational speeds, requiring VAD design to avoid excessive pump flow at minimum operational speed. Further comparison of the same VAD implanted in RV or RA showed that RA implantation achieves better right ventricular unloading due to the higher diastole pump flow. However, compared to RV implantation, RA implantation results in slightly lower *PP*_*pa*_ and pump flow pulsatility.

Regardless of whether in LHF or RHF, during myocardial recovery scenario, total *CO* increases despite the pump flow decreases, indicating better organ perfusion. Therefore, in clinical practice, it is essential to monitor the cardiac function of patients receiving VAD support, particularly in cases of RHF. On one hand, *PAP* elevation must be monitored to prevent potential complications; on the other hand, as right heart function improves and pump flow naturally decreases, anticoagulation strategies should be promptly adjusted to prevent pump thrombosis.

To mitigate hemocompatibility-related complications, numerous studies recommend selecting pumps with flatter H-Q curves to achieve higher PP. Moreover, the enhanced flow pulsatility characteristic—manifested as lower flow during diastole and higher flow during systole—contributes to a reduced risk of suction events and pump thrombosis. A lower thrombosis risk, in turn, allows for reduced anticoagulant dosage, thereby further decreasing non-surgical bleeding events.

Moreover, pumps with flatter H-Q curves exhibit heightened sensitivity to variations in both pre- and after-loads. As a result, they demonstrate slightly inferior ventricular unloading and lower *MAP*, and more prone to backflow when the pressure difference across the pump increases. Although researches have not yet precisely defined the exact effects of this backflow, careful control of the patient’s blood pressure is recommended when utilizing such pumps. However, these limitations can be mitigated by increasing the pump speed to achieve a higher average flow.

**FIGURE 6:**
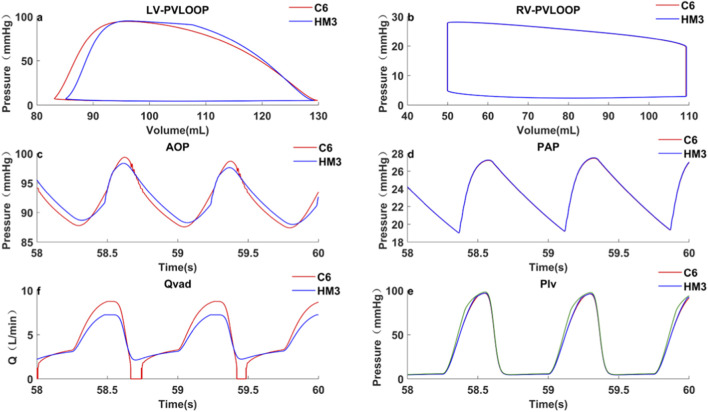
Comparison of hemodynamics between C6 and HM3 under LHF with healthy right heart, at equal MAP, but different average pump flow.

Table 5, Figure 6 illustrates an example from Case 1 where C6 pump speed was increased by 40 rpm. This adjustment resulted in *MAP* and *LVEDV* values equivalent to those achieved by HM3. Notably, the *PP*_*ao*_ remained higher compared to the HM3, while *LVESP* and *LVESV* were lower. This demonstrates that the C6, while maintaining greater pulsatility, can achieve organ perfusion and ventricular unloading comparable to pumps with steeper H-Q curves.

**TABLE 5:** Hemodynamic and pump parameters of C6 and HM3 under LHF with healthy right heart when *MAP* is equal.

Pump	*RPM* */Rpm*	*ΔCO* *L/min*	*MAP* */mmHg*	*PP*_*ao*_ */mmHg*	*CCP* */mmHg*	*LVESV* */ml*	*LVESP* */mmHg*	*LVEDV* */ml*	*Q*_*av*_ */L/min*	*Q*_*VAD,AVG*_ */L/min*	*Q*_*VAD*_ *(PI*_*VAD*_ *)* */L/min*	*MPAP* */mmHg*
C6	3200	1.0	89.6	11.7	81.2	84.0	94.9	131.7	0.2	4.3	0–8.7(2.0)	22.4
HM3	5200	1.1	90.4	8.9	83	84.9	94.9	128.9	0.3	4.3	2.4–7.2(1.1)	22.5
C6	3240	1.1	90.4	10.4	82.7	82.3	93.7	128.9	0.1	4.5	0–8.71(1.91)	22.5

C6: Corheart 6; HM3: HeartMate 3; *ΔCO*, denotes the increment of cardiac output compared with the baseline (L/min); *MAP*: mean aortic pressure (mmHg); *PPao:* aortic pulse pressure (mmHg); *CCP*: coronary perfusion pressure (mmHg); *LVESV*: left ventricular end-systolic volume (mL); *LVESP*: left ventricular end-systolic pressure (mmHg); *LVEDV*: left ventricular end-diastolic volume (mL); *Q*_*av*_: aortic valve flow over one cardiac cycle (L/min); *Q*_*VAD,AVG*_: mean pump flow over one cardiac cycle (L/min); *Q*_*VAD*_ *(PI*_*VAD*_ *)*: pump real-time flow (pump flow pulsatility index); *MPAP*: mean pulmonary artery pressure (mmHg).

**FIGURE 7:**
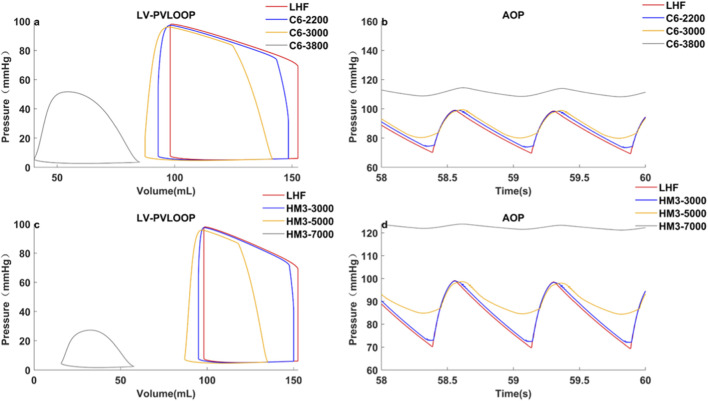
Left ventricular P-V loop and aortic pressure of C6 and HM3 at different speeds under LHF baseline.

It should be noted that the relationship between speed elevation and hemodynamic improvement is not linear, as shown in Figure 7. Regardless of the H-Q curve type, the same magnitude of speed adjustment yields varying effects on hemodynamic parameters, depending on the pre-adjustment operating speed.

A notable and specific dynamic scenario is the acute preload change caused by postural variation. In this situation, pumps with a flatter H-Q curve demonstrate the capability to generate a greater diastolic flow, thereby maintaining effective perfusion more efficiently during abrupt changes in preload. However, this characteristic simultaneously implies a higher risk of suction. Other dynamic clinical conditions, including physical exercise, acute pain, and transient arrhythmias, may likewise induce abrupt alterations in preload and/or afterload. These conditions will be systematically investigated in future work.

Our findings regarding the pulsatility and unloading performance associated with the H-Q curve are consistent in trend with prior *in vitro* and computational analyses of earlier VAD devices. However, the contribution of this study extends beyond these qualitative comparisons by providing the first systematic quantitative hemodynamic analysis of the two mainstream third-generation magnetic levitation centrifugal pumps (Corheart 6 and HeartMate 3). Furthermore, this work addresses critical gaps in the existing literature through detailed analyses of clinically relevant scenarios that have previously lacked systematic comparative assessment, including the systematic comparison of RA versus RV implantation modes in RHF scenarios, and the assessment of VAD performance during the myocardial recovery stage. Finally, by quantifying the hemodynamic response of both designs under acute transient conditions (e.g., acute preload shifts), we enhance the understanding of the H-Q curve’s influence on clinical applications.

## Limitation

This study has several limitations, which may lead to discrepancies between simulation results and actual clinical outcomes. Firstly, this model does not incorporate any physiological autoregulatory mechanisms, such as the baroreflex, which may affect the absolute predicted values of mean aortic pressure and pulmonary pressure, particularly in highly dynamic scenarios like the myocardial recovery phase. Our analysis therefore focuses on the relative differences and trends in performance between the Corheart 6 and HeartMate 3 VADs under the controlled, fixed baseline conditions, rather than aiming for perfect predictive accuracy of the absolute systemic pressures. Secondly, the current model simplifies the complex fluid dynamics within the pump and the ventricle. The pulsatility index of VAD support (*PI*_*VAD*_) is used as a surrogate for flow-related thrombosis. While effective for comparative purposes, this do not fully capture the detailed shear stress and flow separation effects that contribute to thrombosis and hemolysis *in vivo*. In future work, we will incorporate physiological feedback model and couple the lumped parameter model with detailed Computational Fluid Dynamics (CFD) simulations in the pump and cardiac chambers. Furthermore, a significant limitation of our HM3 simulation is the exclusion of artificial pulse. The HM3’s artificial pulse is non-synchronous, and the resultant hemodynamic effect would be highly dependent on the heart rate baseline, a complex dynamic variable whose detailed interaction with the control algorithm is not the central focus of this paper.

## Conclusion

This study employed a numerical model of the circulatory system to investigate the hemodynamic characteristics of VADs with varying H-Q curve slopes across four distinct clinical scenarios. The findings indicate that under conditions of equal average pump flow, pumps with flatter H-Q curve exhibit lower diastolic pump flow and higher systolic pump flow. This generates greater pump flow pulsatility and a higher arterial PP, which can help reduce the risk of vascular malformations, as well as pump suction and pump thrombosis. However, their overall ventricular unloading and organ perfusion is slightly diminished. From a computational perspective, the results suggest that when utilizing pumps with flatter H-Q curves, maintaining a slightly higher average pump flow target may be beneficial to ensure adequate organ perfusion and ventricular unloading while preserving pulsatility.

In contrast to the static condition, during dynamic events such as acute preload reduction caused by postural changes, pumps with flatter H-Q curve exhibit improved preservation of systemic perfusion pressure, albeit at the expense of a greater reduction in LVEDV.

When applying the two types of H-Q curve pumps in RHF scenarios, the characteristic hemodynamic changes observed with RV implantation are analogous to those seen with LV implantation. Conversely, with RA implantation, both pump types exhibit similar hemodynamic performance due to the minimal preload variations. Comparing the results of implanting the same VAD in RA versus RV, RA implantation yields superior right ventricular unloading. However, this comes at the cost of lower pulmonary artery PP and reduced pump flow pulsatility. Furthermore, as cardiac function recovers from HF, an increase in PAP and a decrease in pump flow are observed. Consequently, meticulous attention to anticoagulation management is crucial.

## Data Availability

The original contributions presented in the study are included in the article/supplementary material, further inquiries can be directed to the corresponding authors.
